# The ketogenic diet and hypoxia promote mitophagy in the context of glaucoma

**DOI:** 10.3389/fncel.2024.1409717

**Published:** 2024-05-22

**Authors:** Autumn B. Morgan, Yan Fan, Denise M. Inman

**Affiliations:** Department of Pharmaceutical Sciences, North Texas Eye Research Institute, University of North Texas Health Science Center, Fort Worth, TX, United States

**Keywords:** mitophagy, glaucoma, hypoxia, PGC1 α, BNIP, retinal ganglion cell, Müller glia

## Abstract

Mitochondrial homeostasis includes balancing organelle biogenesis with recycling (mitophagy). The ketogenic diet protects retinal ganglion cells (RGCs) from glaucoma-associated neurodegeneration, with a concomitant increase in mitochondrial biogenesis. This study aimed to determine if the ketogenic diet also promoted mitophagy. MitoQC mice that carry a pH-sensitive mCherry-GFP tag on the outer mitochondrial membrane were placed on a ketogenic diet or standard rodent chow for 5 weeks; ocular hypertension (OHT) was induced via magnetic microbead injection in a subset of control or ketogenic diet animals 1 week after the diet began. As a measure of mitophagy, mitolysosomes were quantified in sectioned retina immunolabeled with RBPMS for RGCs or vimentin for Müller glia. Mitolysosomes were significantly increased as a result of OHT and the ketogenic diet (KD) in RGCs. Interestingly, the ketogenic diet increased mitolysosome number significantly higher than OHT alone. In contrast, OHT and the ketogenic diet both increased mitolysosome number in Müller glia to a similar degree. To understand if hypoxia could be a stimulus for mitophagy, we quantified mitolysosomes after acute OHT, finding significantly greater mitolysosome number in cells positive for pimonidazole, an adduct formed in cells exposed to hypoxia. Retinal protein analysis for BNIP3 and NIX showed no differences across groups, suggesting that these receptors were equivocal for mitophagy in this model of OHT. Our data indicate that OHT and hypoxia stimulate mitophagy and that the ketogenic diet is an additive for mitophagy in RGCs. The different response across RGCs and Müller glia to the ketogenic diet may reflect the different metabolic needs of these cell types.

## Introduction

Glaucoma is a group of chronic optic neuropathies defined by the progressive loss of retinal ganglion cells (RGCs) and the degeneration of their axons, which form the optic nerve. Two of the major risk factors associated with this disease include age and increased intraocular pressure (IOP), with the latter being the only modifiable risk factor. Modern treatment is targeted toward lowering IOP, but this only slows progression and often still leads to blindness. An evaluation of lifetime risk of blindness in open-angle glaucoma examined the incidence of blindness in 592 patients, revealing that ~1 out of 6 glaucoma patients were bilaterally blind and approximately 40% were unilaterally blind despite treatment (Peters et al., 2013). Furthermore, IOP-lowering drugs are unsuccessful at treating glaucoma in 30% of patients, revealing that some cases of glaucoma may be IOP-independent (Doucette et al., 2015). This highlights the need for disease-modifying therapies to address the relentless progression of this disease.

The retina is one of the most metabolically active tissues in the body (Casson et al., 2021). Retinal ganglion cells (RGCs) and their axons, in particular, are sensitive to metabolic dysfunction (Inman and Harun-Or-Rashid, 2017; Casson et al., 2021). Several studies using DBA/2 J mice, a model used to study chronic, age-related glaucoma, have demonstrated early metabolic vulnerability (Williams et al., 2017; Harun-Or-Rashid et al., 2018). Age-related decreases in nicotinamide adenine dinucleotide (NAD^+^) and glutathione are thought to sensitize these neurons to glaucomatous pathology. Additionally, increased levels of the hypoxia-induced transcription factor HIF-1α across retinal neurons and glia support the idea that RGCs undergo metabolic stress before overt glaucomatous progression (Williams et al., 2017). Elevated IOP can alter retinal pyruvate and glucose, exacerbating the age-related metabolic changes (Harder et al., 2020). A significant decrease in adenosine triphosphate (ATP) levels as a result of age and elevated IOP in the optic nerve, as well as a correlation between the rate of axonal dysfunction and the depletion of energy substrates as a result of oxygen–glucose deprivation, further implicate metabolism in glaucoma pathogenesis (Baltan et al., 2010).

Mitochondria are double membrane-bound organelles responsible for the breakdown of energy substrates via oxidative phosphorylation to produce ATP. Nicotinamide adenine dinucleotide (NAD^+^) is an important electron carrier responsible for the maintenance of glycolysis and the electron transport chain (ETC) and has been implicated in regulation of mitochondrial metabolic function and oxidative response (Zhang et al., 2020). Pyruvate, on the other hand, is an end product of glycolysis that becomes oxidized in the tricarboxylic acid (TCA) cycle within mitochondria, ultimately feeding into oxidative phosphorylation. The neuroprotective effect of co-factors like NAD^+^ and substrates like pyruvate directly connects mitochondrial function to the pathology of glaucoma. Oxidative stress through the generation of reactive oxygen species (ROS), mitochondrial DNA mutations (Abu-Amero et al., 2006), and defective mitochondrial quality control mechanisms through IOP-induced fission and cristae depletion (Ju et al., 2008) represent mitochondrial dysfunction that has been implicated in glaucoma (Zhang et al., 2023). In light of this evidence, one avenue for glaucoma treatment could restore metabolic balance through maintenance of mitochondrial function.

Dietary intervention has addressed metabolic dysfunction in a number of different diseases. The ketogenic diet, typically consisting of 70% fat, 20% protein, and 10% carbohydrate, has been used in the treatment of intractable epilepsy since the 1920s, due to seizure suppression by increasing neural inhibition through the adenosine A1 receptor (D’Andrea Meira et al., 2019; Pietrzak et al., 2022). More recently, this diet has gained popularity as an intervention for neurological disorders like Alzheimer’s and Parkinson’s diseases for its ability to serve as an alternative fuel source for brain metabolism, reduce pro-inflammatory responses and oxidative stress, and improve mitochondrial function and biogenesis (Pietrzak et al., 2022). We demonstrated that glaucoma-associated metabolic dysfunction in DBA/2 J mice could be reversed with the ketogenic diet through upregulation of monocarboxylate transporters, increased mitochondrial biogenesis, higher antioxidant response, and reduction of inflammation (Harun-Or-Rashid et al., 2018; Harun-Or-Rashid and Inman, 2018). Mitochondrial homeostasis includes balancing the production of mitochondria with their destruction/recycling. The mitochondrial biogenesis we observed with the ketogenic diet prompted our current exploration of the diet’s effect on mitophagy in glaucoma.

Mitophagy is a specialized category of autophagy that removes damaged or extraneous mitochondria (Chen and Chan, 2009). This occurs through selective uptake into autophagosomes that deliver mitochondria to lysosomes for degradation (Ding and Yin, 2012). Mitochondria can be marked on the surface of their membrane for uptake by the autophagosome through molecular signals like ubiquitin or through receptor-mediated signals from transmembrane proteins (Uoselis et al., 2023). Impaired mitophagy can lead to excessive buildup of dysfunctional mitochondria, leading to increased ROS production, which has been implicated in numerous neurodegenerative diseases and is a hallmark of aging (Shefa et al., 2019; De Gaetano et al., 2021). Mitophagy serves as a critical quality control mechanism for mitochondria, with biogenesis as an important counterweight to maintain mitochondrial number. Excessive mitophagy with lower levels of biogenesis can burden the remaining mitochondria with too high energy production, thereby leading to excessive ROS and ultimately cell death (Schofield and Schafer, 2020). Decreased mitophagy can lead to the accumulation of damaged mitochondria, which also increases ROS (Palikaras et al., 2015). In models of glaucoma, early increases in mitophagy can combat cellular stress from damaged mitochondria, but over time, mitophagy appears to fail, resulting in increased damage to RGCs (Jiménez-Loygorri et al., 2023). Methods to promote mitophagy are neuroprotective. For example, knockout of mitochondrial uncoupling protein 2 (UCP2) promotes mitophagy and decreases RGC loss after ocular hypertension (OHT) (Hass and Barnstable, 2019). Overexpression of parkin, an E3 ubiquitin ligase, can promote mitophagy and protect RGCs in glaucoma (Dai et al., 2018). Several genes associated with glaucoma pathology encode various players in the mitophagy pathway, such as fusion proteins mitofusin-2 (MFN-2) and optic atrophy-1 (OPA-1), which regulate mitochondrial fusion (Hu et al., 2018); TANK-binding kinase 1 (TBK1), which phosphorylates autophagy receptors (Richter et al., 2016); and optineurin (OPTN), a mitophagy receptor with mutant isoforms observed in glaucoma patients (Rezaie et al., 2002). Thus, mitophagy is a mitochondrial quality control mechanism with important implications for glaucoma.

HIF-1α is a transcription factor that is stabilized under low oxygen (hypoxic) conditions and whose activity leads to the transcription of several metabolism genes such as hexokinase and glucose transporter-1 (Semenza, 2011). Several studies demonstrated the presence of hypoxia after ocular hypertension that led to HIF-1α activation (Tezel and Wax, 2004; Chidlow et al., 2017; Jassim and Inman, 2019; Jassim et al., 2022), which altered retinal metabolism and disrupted nucleus-to-mitochondria communication (Jassim et al., 2021). Our lab has shown that chronic HIF-1α stabilization, in the absence of glaucoma, can result in upregulation of mitophagy as measured by increased mitolysosome number and increased protein levels of BCL2 Interacting Protein 3 (BNIP3) (Nsiah et al., 2023). BNIP3 has been associated with hypoxia-associated mitophagy because it is a HIF-1α target (Bellot et al., 2009; He et al., 2022), and its phosphorylation allows it to bind microtubule-associated protein 1A/1B-light chain 3 (LC3), the adaptor protein that recruits cargo to the autophagosome (Lee and Lee, 2016). Having observed incidents of hypoxia in the context of glaucoma, we sought to directly test whether hypoxic cells upregulate mitophagy. For this purpose and for our test of the impact of the ketogenic diet on mitophagy, we used the MitoQC transgenic mouse developed by McWilliams et al. (2016) along with semi-automatic quantification tools developed specifically for use with this model (Montava-Garriga et al., 2020).

## Materials and methods

### Animals

The C57BL/6-Gt(ROSA)26Sor^tm1^^(CAG-mCherry/GFP)Ganl/GanlH^ mice were obtained from the MRC Harwell Institute, which distributes this strain on behalf of the European Mouse Mutant Archive (EMMA: www.infrafrontier.eu). The repository number is EM:11343. The C57BL/6-Gt(ROSA)26Sor^tm1^^(CAG-mCherry/GFP)Ganl/GanlH^ mice were originally produced from transgenic mice generated using Taconic Artemis (McWilliams et al., 2016) using ES cells from C57Bl/6NTac mice. Once at the University of Dundee, the mice were backcrossed to the C57Bl/6 J strain for more than 10 generations before distribution (I. Ganley, personal communication). Mice were housed at the University of North Texas Health Science Center and are hereafter referred to as “MitoQC” mice. The MitoQC mice were generated by inserting a CAG promoter cassette with an open reading frame for the mCherry-GFP-FIS1 fusion protein and Kozak sequence (GCCACC) into the mouse Rosa26 locus, which were maintained through a C57BL/6 J background (McWilliams et al., 2016). The fusion of mCherry-GFP to the outer mitochondrial membrane protein allows the mitochondrial network to fluoresce green and red under steady-state conditions (McWilliams et al., 2016). The GFP fluorescence is pH-dependent; therefore, when the mitochondria are delivered to the lysosomes, the GFP fluorescence is quenched due to the acidic microenvironment, leaving only the mCherry fluorescence, thereby indicating mitophagy (McWilliams et al., 2016).

### Diet

Mice within the ketogenic diet experimental groups were switched to a low-carbohydrate ketogenic diet (Research Diets, catalog # D12369B) for 5 weeks, which consisted of 10.4% protein, 0.1% carbohydrate, and 89.5% fat. The two control diet groups continued to eat the standard lab chow (Diet 5,058, PicoLab Mouse Diet 20), which consisted of 23.2% protein, 55.2% carbohydrate, and 21.6% fat. The standard lab chow is pellets that were placed in the cage hopper, while the ketogenic diet is a soft dough placed in a small stainless-steel bowl. Mice were weighed before the experiment, then on days 3, 7, 10, 13, 17, 20, 30, and 35. The food was weighed on the same schedule to monitor diet consumption. A Nova Max Plus hand-held ketone testing device was used to measure ketones from tail vein blood once weekly for three mice on the ketogenic diet. Mice were split into four groups: control diet (Naive, *n* = 14), Control diet with ocular hypertension (OHT, *n* = 14), Ketogenic diet (KD, *n* = 13), and Ketogenic diet with OHT (KD + OHT, *n* = 13).

### Acute glaucoma model

To increase intraocular pressure, mice were anesthetized with isoflurane (2.5%) delivered through a vaporizer with oxygen, then received a drop of 0.25% tropicamide (Sandoz Inc.) on the cornea to dilate the pupil, followed by 0.5% proparacaine hydrochloride eye drops (Akorn) for local pain relief. A glass-pulled micropipette was used to inject 2 μL of 8-μm-diameter magnetic beads (Bangs Laboratories, UMC4F Compel) into the anterior chamber while curved forceps stabilized the eye. A neodymium magnet was used to draw the beads into the iridocorneal angle to occlude the trabecular meshwork while the micropipette was still in place. Once the beads were in place, the micropipette was removed, and Pura-lube ophthalmic ointment was applied to the eye while the mice were placed on a warm water blanket to recover.

### Intraocular pressure

For intraocular pressure measurements, mice were anesthetized with isoflurane as described above, while a Tono-lab rebound tonometer (calibrated for mice; iCare) was used to take 10–20 measurements to calculate an average. The instrument-calculated running average was only used to determine the quality of the measurements through the provided standard deviation. All IOP measurements were recorded within 3 min of anesthesia to avoid anesthesia-associated IOP decline. Baseline IOP was taken prior to ocular hypertension, then every 7 days after the procedure for 4 weeks. An IOP integral was calculated by subtracting the baseline IOP from the weekly average, multiplying the difference by the number of days between measurements, and summing the week-by-week numbers. IOP integrals are expressed in mmHg-days.

### Ischemia/reperfusion

Mice were anesthetized with 2.5% isoflurane and placed on a heating pad for thermal regulation; body temperature was maintained at 37°C. Eyes were topically anesthetized with 0.5% proparacaine hydrochloride (Akorn), and pupils were dilated with 0.5% tropicamide. A 32G needle connected to a reservoir filled with sterile phosphate-buffered saline (PBS) was inserted into the anterior chamber (Dibas et al., 2018). The reservoir was elevated to increase the IOP to 40 mmHg above systolic blood pressure for 30 min. After the IOP increase, the needle was removed to allow for reperfusion. Mice treated in this way served as a positive control for hypoxia analysis. Two additional groups were included in the pimonidazole experiment: Control and Control + OHT mice. Mice in the Control + OHT group (*n* = 3) were induced with elevated IOP using the magnetic microbead model, as described in the acute glaucoma model section above, 24 h before pimonidazole (Pimo) injection. Mice from the Control group (*n* = 3) underwent 15 min of isoflurane anesthesia 24 h before injection to control for any effect of anesthesia on hypoxia. Hypoxia was identified using pimonidazole hydrochloride (Hypoxyprobe Pacific Blue, Hypoxyprobe, Burlington, MA, United States), a compound that forms adducts in cells that experience a partial pressure of oxygen <10 mmHg. Pimonidazole hydrochloride (60 mg/kg) was diluted in sterile PBS and injected IP 10 min after completion of the IOP increase; mice were sacrificed 90 min later using an overdose of sodium pentobarbital (Euthasol) and perfused first with 0.1 M PBS (pH 7.4) then with 4% paraformaldehyde (Jassim and Inman, 2019). Retinas were cryoprotected in 30% sucrose and then processed as described in the section on Tissue Collection. Retinal sections were immunolabeled with anti-RBPMS antibody and an antibody against pimonidazole (Pimo) that was conjugated to Pacific Blue fluorophore, then cover-slipped with Fluoromout G (Southern Biotech) for imaging. Mitolysosome quantification was performed on Pimo-positive cells as described under Mitolysosome Quantification (below), with the exception that the blue channel analyzed was Pimo, not RBPMS. Quantification of Pimo-positive RGCs was done on images cropped in ImageJ to include only the retinal ganglion cell layer.

### Tissue collection

Mice from each treatment group (4–6 animals) were euthanized through an overdose of sodium pentobarbital (300 mg/kg, Euthasol) via IP injection, followed by transcardial perfusion with 0.1 M PBS, then 4% paraformaldehyde (PFA) for tissue fixation. Whole eyes were extracted, fixed in 4% PFA for 30 min, and then cryoprotected in 30% sucrose with 0.02% sodium azide overnight. Lenses were removed from the globes, then the globes were embedded in OCT and frozen in liquid nitrogen-cooled isopentane. Retinas were sectioned using a cryostat at 10 μm and then used for immunohistochemistry. The remaining mice from each group (8–10) were euthanized via carbon dioxide, followed by cervical dislocation. Eyes were removed by forceps and then dissected. The retina was removed, placed in a tube, and then flash frozen in liquid nitrogen until downstream mRNA and protein analysis.

### Immunohistochemistry

During the first day of immunohistochemistry, tissues were washed using 0.1 M PBS, incubated in blocking solution (5% donkey serum, 0.5% Triton X-100 in 0.1 M PBS) for 1 h, then incubated in primary antibody diluted with the blocking solution at 4°C for 24 h. On day 2, tissues were washed again using 0.1 M PBS, incubated in secondary antibody diluted by blocking solution for 2 h at room temperature, covered to prevent light exposure, washed again, and cover-slipped using DAPI Fluoromount G (Southern Biotech) or Fluoromount G (Southern Biotech). Primary antibodies used are listed in Table 1. The secondary antibodies used were anti-rabbit Alexa Fluor 647 (1:250, 711–605-152, Jackson ImmunoResearch), anti-Chicken Alexa Fluor 647 (1:250, 703–605-155, Jackson ImmunoResearch), anti-Rabbit Dylight 405 (1:250, 711–475, Jackson ImmunoResearch), and anti-Mouse Alexa Fluor 647 (1:250, 715–605-150, Jackson ImmunoResearch). Secondary antibodies were raised in donkeys against the species appropriate to the primary antibody.

**Table 1:** Antibodies used in the study.

Antigen	Species	Manufacturer	Catalog number	Dilution
RBPMS	Rabbit	GeneTex	GTX118619	1:250 IF
RBPMS	Mouse	Novus Biologicals	OTI3B7	1:250 IF
Vimentin	Chicken	Novus Biologicals	NB300-223	1:250 IF
Pimonidazole	Rat	Hypoxyprobe	hp15-100kit	1:200 IF
PGC1α	Rabbit	Novus Biologicals	NBPI-04676	1:300 IF, 1:50 CE
BNIP3	Rabbit	Cell Signaling	3769S	1:250IF, 1:25 CE
BNIP3L (NIX)	Rabbit	Cell Signaling	12396S	1:250IF, 1:100 CE

CE, capillary electrophoresis; IF, immunofluorescence.

### Microscopy

Images for RGC and Müller glia mitolysosome quantification were taken at 63× magnification on an inverted Leica DMi8 Confocal microscope. Two images were taken from each retinal section across six retinal sections per eye in each group (24 images total per mouse). For mitolysosome quantification with pimonidazole staining, images were taken at 63× magnification on a Zeiss LSM 880 confocal with 12 images total per eye from each group (24 images per mouse). Retinal sections immunolabeled with PGC-1α, BNIP3, or NIX and cover-slipped with Fluoromount G for labeling density analysis were also imaged on the Zeiss LSM 880 Confocal at 20× magnification with 12 images per eye in each group (24 images total per mouse). RGC quantification images were taken on a Leica DM4 upright fluorescent microscope using StereoInvestigator (MicroBrightfield Biosciences) at 20× magnification with two peripheral and two central images from each retinal section across a total of six retinal sections (24 images) for four eyes from each group. All representative mitolysosome images were taken at 63× magnification with 1.6× zoom on the same Zeiss confocal, then processed with deconvolution using Huygens software (Scientific Volume Imaging).

For immunolabeling analysis, we used an ImageJ macro that quantifies the pixels of a region of interest traced within a retinal section after thresholding for background that is established within each section (Dengler-Crish et al., 2014). RGCs were also quantified manually from retinal sections immunolabeled for RBPMS using regions of interest traced in ImageJ.

### Mitolysosome quantification

Retinal sections immunolabeled with RBPMS or vimentin and cover-slipped with DAPI were used to quantify mitolysosomes in the retinal ganglion cells and Müller glia. A Fiji-ImageJ macro called “mitoQC Counter” was used, with guidance from Montava-Garriga et al. (2020). This process involved splitting the color channels to allow for the selection of the channel representing Vimentin or RBPMS. After adjusting the brightness/contrast, a median filter with a radius of 5.0 pixels was applied. Next, the immunolabeled cells underwent thresholding before particle analysis for a set size of 0.01 infinity. Finally, once the channels were merged back into the appropriate colors, the mQC_counter plugin was selected with green channel assigned to 2, red channel assigned to 1, and ratio threshold set at 0.5. From there, the mitolysosome count was normalized to the cell size given by the plugin.

### Protein analysis

Flash frozen retinas were homogenized into protein by adding 100 μL of T-PER (Tissue Protein Extraction Reagent, Thermo Fisher Scientific Product # 78510) with 1 μL of 100× Halt Protease & Phosphatase Inhibitor (Thermo Fisher Scientific, #1861280), then pulling through a 21G needle to shred the tissue before centrifugation for 15 min at 10,000 *g* at 4°C. The supernatant was collected into a new tube and used for protein analysis. Total retinal protein was determined through the use of Pierce^™^ BCA Protein Assay Kit (Thermo Fisher Scientific REF# 23225) with absorbance read using a Cytation 5 (Biotek) plate reader to allow data to be normalized by total protein level in each retinal sample. Protein analysis was performed via capillary-based electrophoresis immunoassay using the Protein Simple Jess instrument (Biotechne). Proteins analyzed and antibody concentrations are listed in Table 1 with CE designation.

### mRNA analysis

mRNA was extracted from flash frozen retinas using a Qiagen kit (CAT# 74134). The isolated mRNA was then transformed into cDNA using the Verso cDNA Synthesis Kit (Thermo Fisher Scientific, AB-1453/B) for stable storage and analyzed using real-time quantitative qPCR with a QuantStudio 5 real-time PCR System instrument (Applied Biosystems). TaqMan assay probes utilized in mRNA analysis included *Ppargc1a*, Cat. No. Mm01208835_ml, and *Actb*, Cat. No. Mm01205647_g1 (Thermofisher). *β-actin* was used as the housekeeping gene.

### Statistical analysis

Statistical analysis was performed with GraphPad Prism version 10 (La Jolla, CA, United States). Tests of normality were employed to inform downstream parametric or non-parametric analyses. For parametric comparisons across groups, one-way ANOVA or two-way ANOVA and Tukey’s multiple comparisons test were used. For non-parametric data, Brown-Forsythe and Welch ANOVA, or mixed effects, and Dunnett’s multiple comparisons tests were used. Only *p* < 0.05 was considered significant. The data are presented as mean ± SD.

## Results

### Ketogenic diet and intraocular pressure

**Figure 1:**
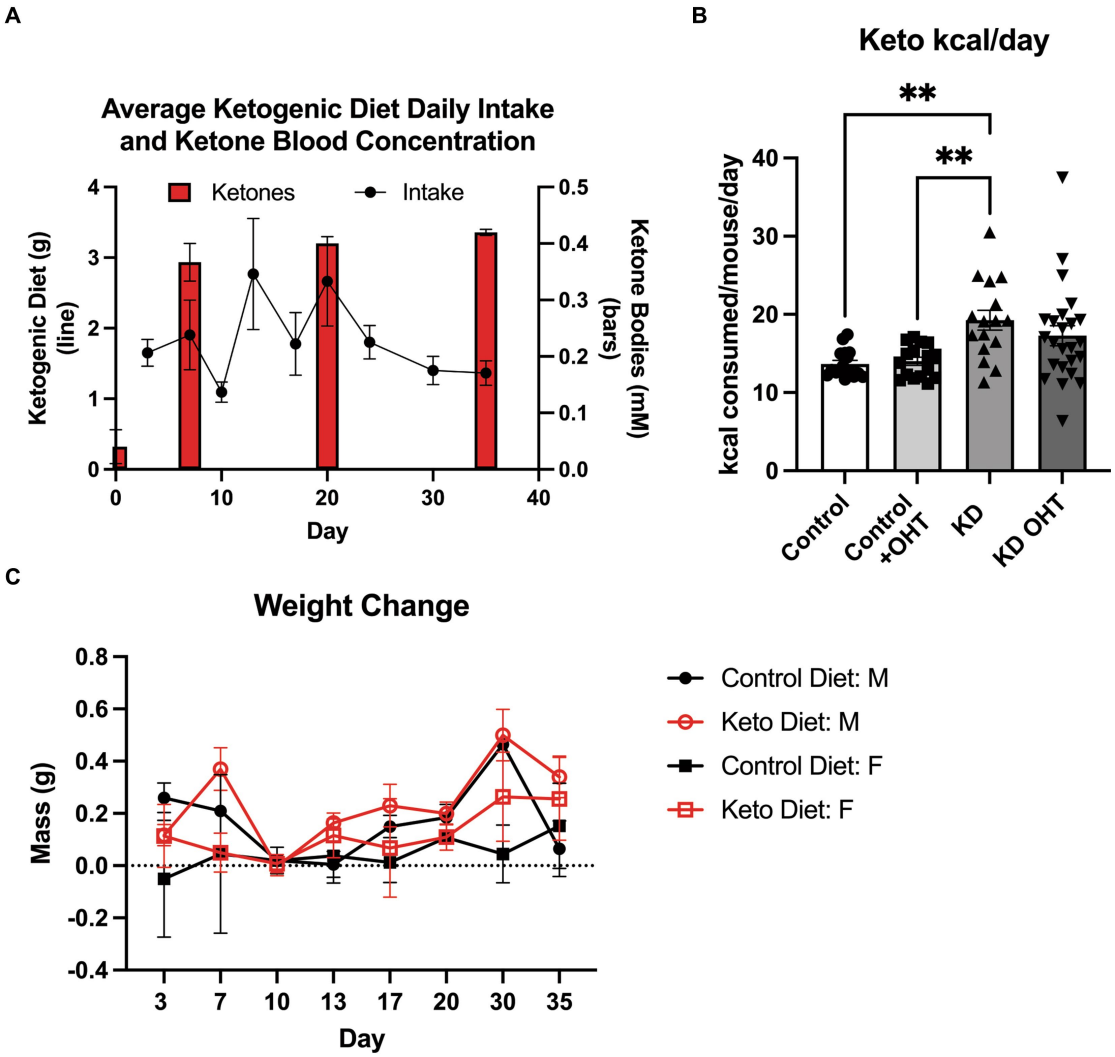
Ketogenic diet. **(A)** Mice were provided *ad libitum* access to the ketogenic diet, and the amount of diet consumed was measured twice weekly. The line graph shows the average consumption (in grams) of the diet per day per cage (*n* = 10). Ketone bodies as measured by blood draw (red bars) demonstrate attainment of ketosis (~0.4 mM) by the end of the first week of the diet. Ketone body levels were significantly elevated by 7 days (*p* < 0.0001; *n* = 3), as determined by one-way ANOVA. Ketosis was maintained throughout the study. For the average ketogenic diet consumption, a two-way ANOVA indicated a significant effect of cage (*p* = 0.0007) and day (*p* = 0.011). **(B)** Kilocalories consumed by the mice per day while on the ketogenic diet. The mice on the ketogenic diet (KD) consumed significantly more kilocalories than either the Control or the Control + OHT groups (**p = 0.0028 and **p = 0.0061, respectively); *n* = 4 for Control and Control + OHT groups; *n* = 6 for KD and KD + OHT groups. There was no difference in kilocalories consumed among the KD + OHT, Control, and Control + OHT groups, as determined by the Kruskal–Wallis test with multiple comparisons. **(C)** Mouse weight change compared to baseline at day 0, plotted over the duration of the study (*n* = 6 for Control; *n* = 9 for KD). No significant weight losses or gains were observed, based on two-way ANOVA.

Mice were placed on the ketogenic diet 1 week prior to induction of ocular hypertension (OHT). Our intention was to acclimate them to the diet and only use those mice who were freely eating it. We found that all MitoQC mice in the ketogenic diet groups consumed the diet; Figure 1A shows the daily average ingestion of the diet in grams charted with the blood concentration of ketones (β-hydroxybutyrate). The average ketogenic diet ingestion was 1.82 ± 64 g per day, much lower than the average amount of standard lab chow (4.19 ± 0.38 g). The high caloric density of the ketogenic diet allowed mice to eat less yet still meet or exceed the kilocalorie per day average for the mice on standard chow (Figure 1B). Mice on the ketogenic diet alone ingested significantly more calories than either the Control (*p* = 0.0028) or Control + OHT (*p* = 0.0061) groups. We also monitored weight gain or loss on the standard lab chow (control) and the ketogenic diet (Figure 1C), finding no significant differences across sex or group. Over the 5 weeks monitored, male mice on the control diet gained an average of 0.17 ± 0.15 g, while females on the control diet gained 0.046 ± 0.06 g. Male mice on the ketogenic diet gained an average of 0.23 ± 0.16 g, while females gained 0.12 ± 0.09 g. There were no significant weight gains or losses across groups.

**Figure 2:**
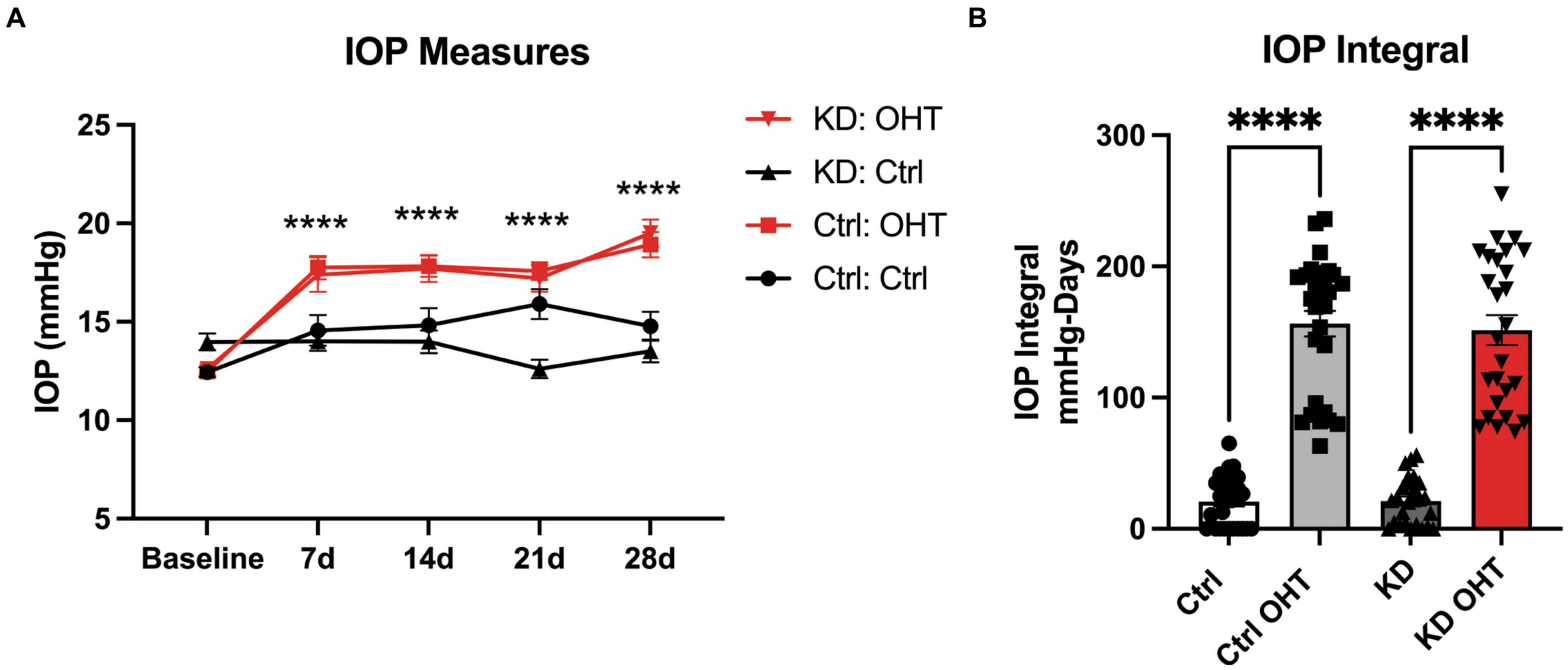
Ocular hypertension model. **(A)** IOP was significantly elevated by 7 days after ocular hypertension (OHT) in the Control + OHT (*n* = 14) and KD + OHT (*n* = 13) groups and remained significantly elevated over the 28 days of the study period (*p* < 0.0001) by the mixed-effects model followed by Dunnett’s multiple comparisons test (Control *n* = 14; KD *n* = 13). **(B)** The IOP integral, or area under the curve for the IOP plot by group, showed a significant increase in IOP integral for the Ctrl + OHT (*n* = 14) and KD + OHT (*n* = 13) groups (*p* < 0.0001); the IOP integral was not statistically different across the Ctrl + OHT and KD + OHT groups, by one-way ANOVA with Tukey’s multiple comparisons (*n* = 14 for Control group; *n* = 14 for KD group).

Intraocular pressure (IOP) was measured for all groups, with mice subjected to OHT showing significantly greater IOP than control groups (*p* < 0.0001), beginning at 7 days and continuing to the end of the experiment at 28 days after OHT induction (Figure 2A). We calculated the IOP integral, the magnitude of the IOP injury, and found that the OHT groups had a significantly higher IOP integral than the Control or Ketogenic Diet (KD) groups (*p* < 0.0001). There was no difference in IOP integral for the Control-OHT group compared to the KD-OHT group (Figure 2B).

### Retinal ganglion cell quantification

**Figure 3:**
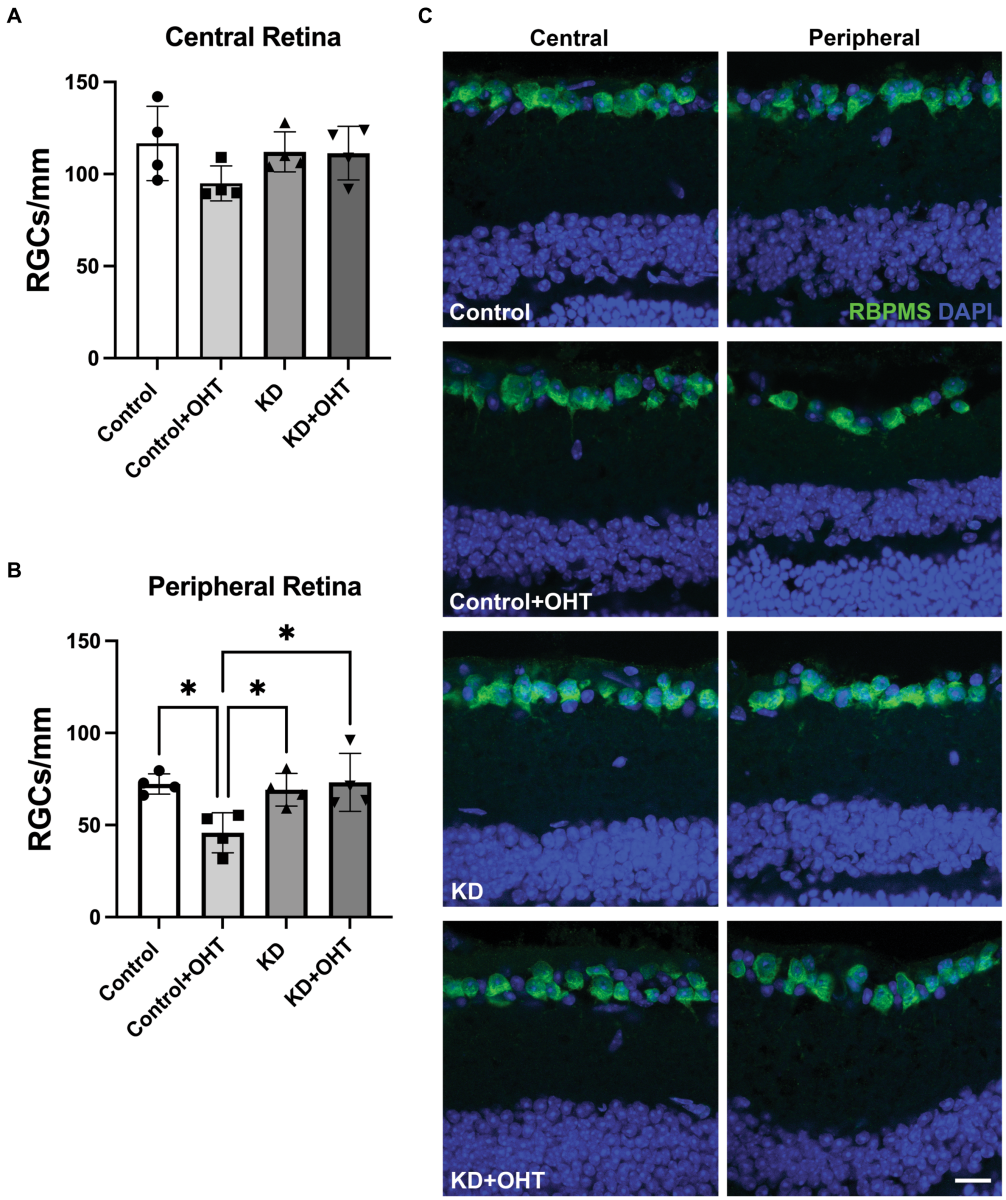
Retinal ganglion cell quantification. **(A)** RBPMS-positive RGCs were counted in retinal sections adjacent to the optic nerve head (central retina). There were no differences in RGC number across groups in the central retina from one-way ANOVA with Tukey’s multiple comparisons. Data represent averages of individual sections from four eyes per group (*n* = 2 for each group). **(B)** RBPMS-positive RGCs quantified in the peripheral retina showed a significant decline in the Control + OHT group compared to the Control (*p* = 0.022), the KD (*p* = 0.045), and the KD + OHT (*p* = 0.018) groups. Data represent averages of individual sections from four eyes per group (*n* = 2 for each group). Statistics from one-way ANOVA with Tukey’s multiple comparisons. **(C)** Representative immunolabeling for RBMPS (green) and nuclear staining with DAPI (blue) using retinal sections taken from the Control, Control + OHT, KD, and KD + OHT groups is shown. Central retinal sections are on the left, and peripheral retinal sections are on the right. Scale bar = 20 μm.

Retinal ganglion cell number in the central retina did not differ across the groups (Figure 3A) for mice subjected to OHT. In the peripheral retina, however, the Control + OHT group had significantly fewer RGCs than the Control (*p* = 0.022), the KD (*p* = 0.045), and the KD + OHT groups (*p* = 0.018), as shown in Figure 3B. The significantly higher numbers of peripheral RGCs in the KD + OHT group versus Control are consistent with our previous study that showed the neuroprotective effect of the ketogenic diet in the DBA/2 J model of glaucoma (Harun-Or-Rashid et al., 2018). Examples of sectioned retina from each group immunolabeled for the RGC marker RBPMS (green) taken from both the central (left) and peripheral (right) retina are shown in Figure 3C.

### Mitolysosome quantification in retinal ganglion cells

**Figure 4:**
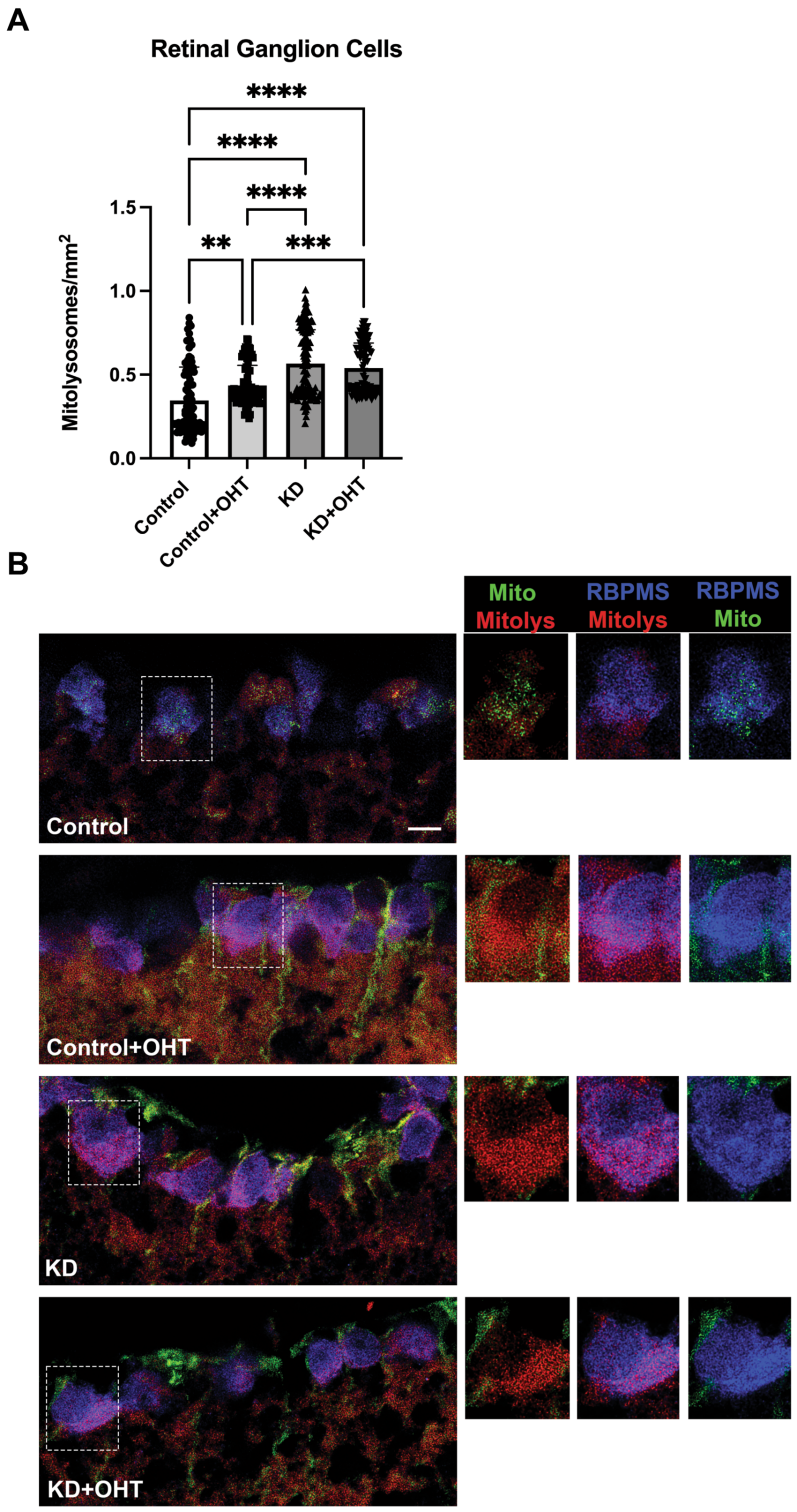
Mitolysosomes quantified in the retinal ganglion cells. **(A)** Ocular hypertension in the control retina (Control + OHT; *n* = 4) resulted in a significant increase in mitolysosomes as compared to the naïve control retina (Control; *p* = 0.0023; *n* = 4). The ketogenic diet (KD; *n* = 6) resulted in a significant increase in mitolysosomes per mm^2^ of cell area compared to Control retina (*p* < 0.0001) and Control + OHT (*p* < 0.0001). There is no difference in mitolysosome numbers between mice on the ketogenic diet with ocular hypertension (KD + OHT; *n* = 6) and the ketogenic diet alone (*p* = 0.64). KD + OHT mice had a significantly greater number of mitolysosomes than Control + OHT mice (*p* = 0.002). Statistics from one-way ANOVA with Tukey’s multiple comparisons test. **(B)** Immunofluorescence of RGCs with RBPMS (blue) in the MitoQC retina (mitochondria are green and red); red immunofluorescence alone corresponds to mitolysosomes. RBPMS-positive RGCs traced in white boxes (insets) are then shown to the left at higher magnification to highlight the mitochondria and mitolysosomes in the RGCs. The boxed insets have labels above to indicate which cellular elements are depicted: mitochondria and mitolysosomes (green and red), RGCs and mitolysosomes (blue and red), and RGCs and mitochondria (blue and green). The Control group has even distribution of green and red puncta, while the Control + OHT, KD, and KD + OHT groups show primary mitolysosomes in the RBPMS-positive RGCs. Scale bar = 20 μm.

Using the ImageJ macro mitoQC_Counter (see Methods), we quantified the mitolysosomes (red puncta) in the RGCs from retinal sections that had been immunolabeled with RBPMS for RGCs. Only the mitolysosomes appearing within RGCs/colocalized with RBPMS were quantified. Figure 4A shows that OHT significantly increased the appearance of mitolysosomes in the RGCs (*p* = 0.0023) of Control + OHT retina compared to Control. The ketogenic diet (KD) also significantly increased mitolysosome number in the RGCs (*p* < 0.0001) compared to Control. Ocular hypertension did not have an additive effect on the increased mitolysosomes from the KD since the KD + OHT group mitolysosome number did not differ from KD alone. Both the KD and KD + OHT groups had significantly more mitolysosomes than the Control + OHT group (*p* < 0.0001 for KD and *p* = 0.0002 for KD + OHT), suggesting that the KD was a more powerful stimulus than OHT for mitolysosome generation in RGCs. Images of mitochondria (green + red fluorescence) and mitolysosomes (red fluorescence) in retinal sections immunolabeled for RGCs (blue) are shown in Figure 4B. Green and red puncta (mitochondria) are evident in the Control group RGCs, but the green fluorescence in the OHT and KD groups is largely confined to the Müller glial processes surrounding the RGCs (Figure 4B, see insets). RGCs in the Control + OHT, KD, and KD + OHT groups are filled with mitolysosomes (red puncta).

### Mitolysosome quantification in Müller glia

**Figure 5:**
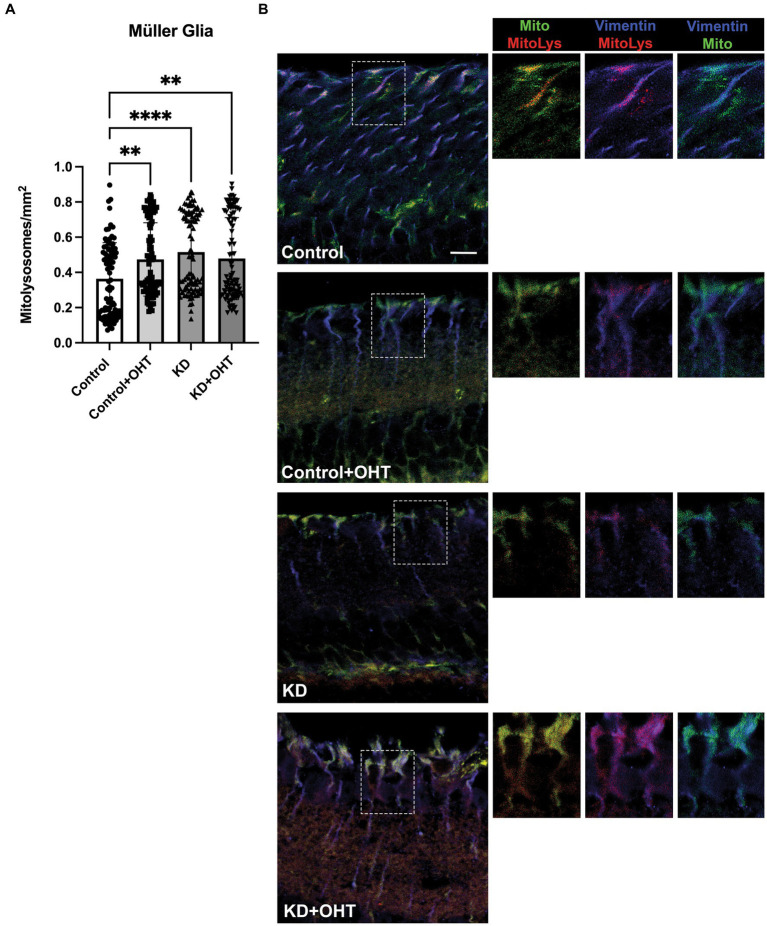
Mitolysosomes quantified in the Müller glia. **(A)** Ocular hypertension in the control retina (Control + OHT; *n* = 4) resulted in a significant increase in mitophagy as compared to the naïve control retina (Control; *p* = 0.0036; *n* = 4). Both the ketogenic diet group (*p* < 0.0001; *n* = 6) and the ketogenic diet with ocular hypertension (KD + OHT, *p* = 0.0022; *n* = 6) had significantly greater mitolysosome number than the Control retina. There was no difference in the mitolysosomes per Müller glia cell in the mice in the Control + OHT, ketogenic diet (KD), and KD + OHT groups. Statistics from one-way ANOVA with Tukey’s multiple comparisons test. **(B)** Immunofluorescence of vimentin labeling for Müller glia (blue) combined with the MitoQC green and red fluorescence of mitochondria. Lone red puncta correspond to mitolysosomes. Each left panel showing blue Müller glia has a traced inset that is then shown at higher magnification on the right, with breakouts of different combinations of fluorescence. The boxed insets have labels above to indicate which cellular elements are included; mitochondria and mitolysosomes (green and red), Müller glia and mitolysosomes (blue and red), and Müller glia and mitochondria (blue and green). The Control + OHT and KD + OHT groups have expanded Müller glia basal processes in which both green and red puncta are observed. Scale bar = 20 μm.

Mitolysosomes quantified in Müller glia showed that OHT and the KD had roughly the same impact on mitolysosome generation since the number of mitolysosomes in the Control + OHT, the KD, and the KD + OHT groups did not differ (Figure 5A). All three groups had significantly greater numbers of mitolysosomes than the Control group (*p* = 0.0036 for Control + OHT, *p* < 0.0001 for KD, and *p* = 0.0022 for KD + OHT). Figure 5B shows MitoQC mouse retinal sections from each of the groups also immunolabeled with vimentin (blue) for Müller glia. All groups show mitochondria (green and red puncta) throughout the Müller glia, with increased expansion of Müller glial basal processes after OHT (see insets for Control + OHT and KD + OHT immunofluorescence, Figure 5B), and greater numbers of mitolysosomes (red puncta) in the Control + OHT, KD, and KD + OHT groups.

### Mitolysosome quantification in hypoxic cells

**Figure 6:**
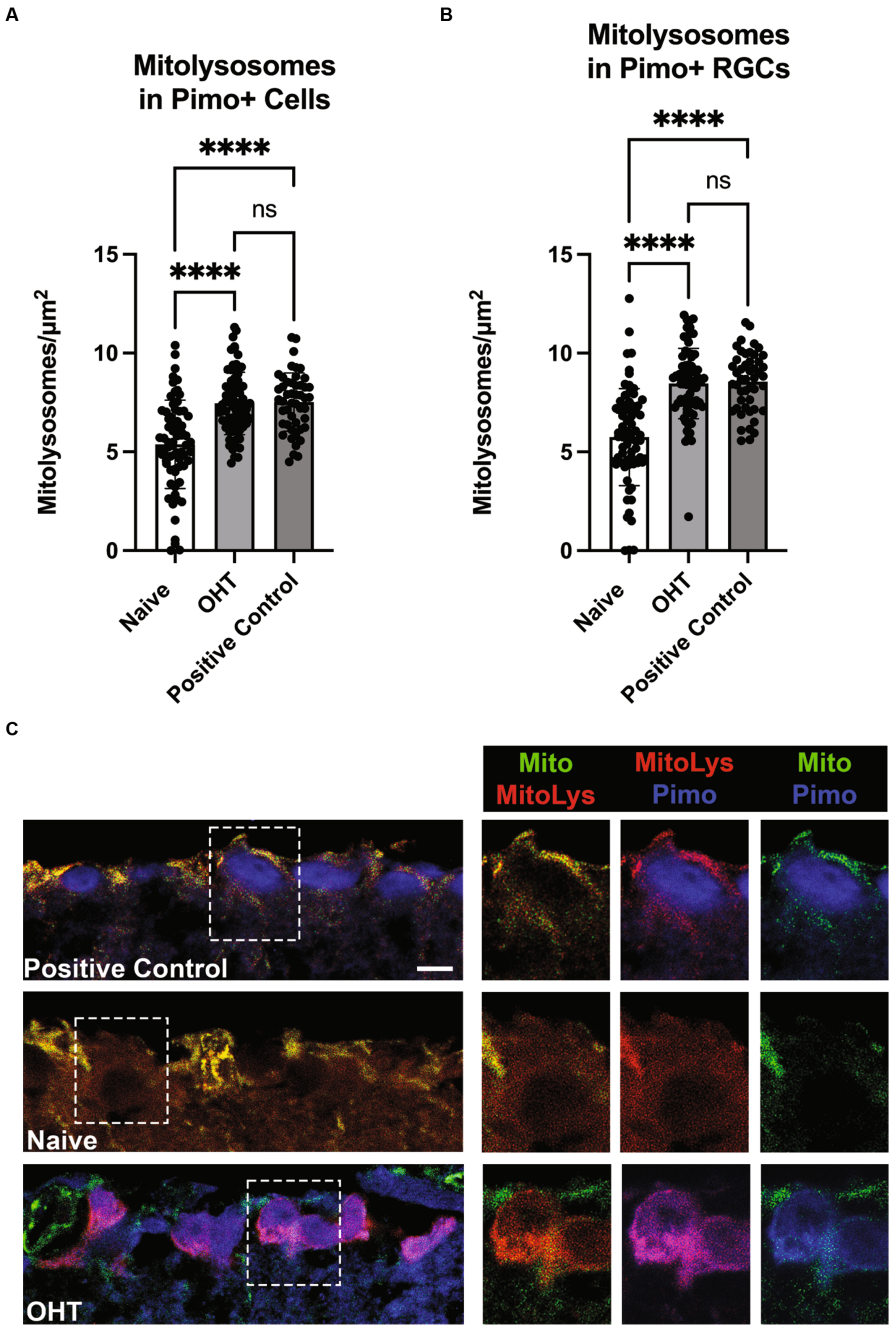
Low oxygen tension as a stimulus for mitophagy. **(A)** Pimonidazole-positive cells in retinal cross-sections from Naive (naïve, *n* = 3) and Naive mice exposed to OHT (OHT, *n* = 3) were examined for their mitolysosome number. A retina exposed to 40 mmHg of ischemia–reperfusion for 30 min (see Methods) served as the Positive Control (*n* = 2). Both the OHT and Positive Control groups had a significantly greater number of mitolysosomes as compared to the Naive group (*p* < 0.0001). The OHT and Positive Control group mitolysosome numbers were not statistically different. Statistics from one-way ANOVA with Tukey’s multiple comparisons. **(B)** Pimonidazole-positive RGCs, as identified by colocalization of pimonidazole and RBPMS, were evaluated for mitolysosome number, with pimonidazole-positive RGCs in OHT (*n* = 3) and Positive Control (*n* = 2) retina showing significantly greater mitolysosome number than Naive retina (*p* < 0.0001; *n* = 3). Similarly to full retina, there was no difference in mitolysosome number in OHT and Positive Control groups. Statistics from Brown-Forsythe and Welch one-way ANOVA with Dunnett’s multiple comparisons. **(C)** Representative pimonidazole labeled retinal sections from MitoQC mice. On the left are ganglion cell layer views of Positive Control, Naive, and OHT retinal sections, with dotted line insets further magnified and shown to the right. Labels indicate the identification of mitochondria (green), mitolysosomes (red), and pimonidazole (blue). Note the absence of pimonidazole labeling in the Naive group ganglion cell layer. In the OHT group, the pimonidazole and mitolysosomes are colocalized, resulting in magenta color for the RGCs in the mitolysosome + pimonidazole inset. Scale bar = 20 μm.

Oxygen tension across retinal layers can vary based on distance from vasculature or the choroid, as well as from circumstances such as OHT (Casson et al., 2021). Previous studies have demonstrated that the IOP elevations experienced by mice exposed to the magnetic microbead model of OHT (Jassim and Inman, 2019; Jassim et al., 2022) or the DBA/2 J model of glaucoma (Jassim et al., 2021) can result in hypoxia in retinal neurons and glia. To test whether low oxygen tension experienced by retinal cells can stimulate mitophagy, we injected Naive mice or those exposed to OHT (OHT) with pimonidazole, a compound that creates covalent adducts in cells that experience a partial pressure of oxygen lower than 10 mmHg (hypoxia). Our positive control was the retina that had been exposed to 40 mmHg of IOP for 30 min. We then evaluated mitolysosome generation in retinal cells or RBPMS-positive RGCs that were pimonidazole-positive. We observed a significant increase in mitolysosomes in pimonidazole (Pimo)-positive cells throughout retina exposed to OHT (Naive vs. OHT, *p* < 0.0001; Figure 6A). Mitolysosome number in the OHT retina did not differ from the Positive Control. Focusing the analysis on RGCs, we observed a significant increase in mitolysosomes in the Pimo-positive and RBPMS-positive RGCs in the OHT group versus Naive (*p* < 0.0001, Figure 6B). Figure 6C shows Pimo labeling in the ganglion cell layer of the Positive Control and the OHT retinal sections. The Naive group shows no Pimo (blue) since no RGCs were Pimo-positive. These data indicate that Pimo-positive RGCs have a significantly higher mitolysosome number than Naive RGCs, suggesting that hypoxia is a stimulus for mitophagy in the retina.

### Evaluation of mitochondrial biogenesis

**Figure 7:**
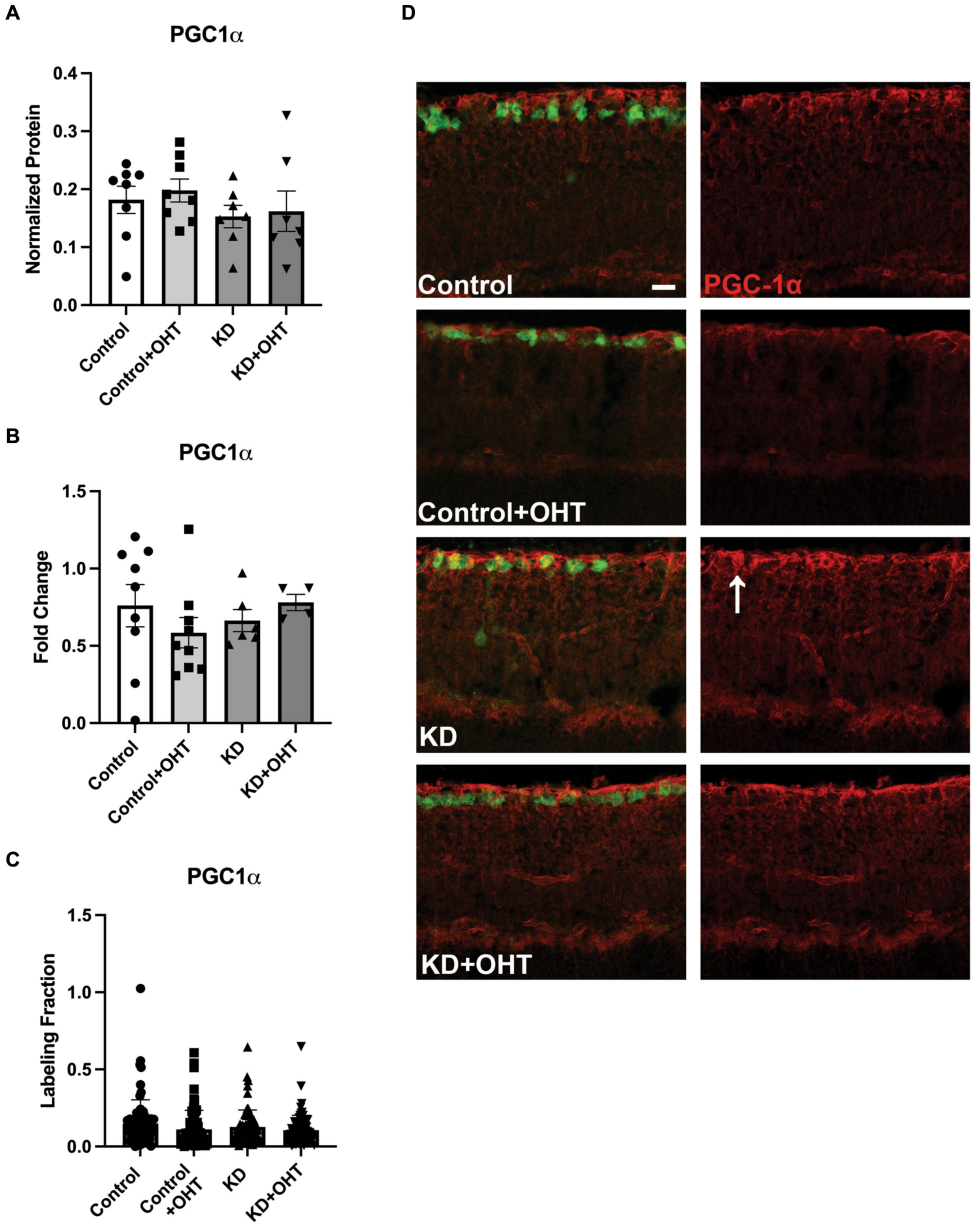
PGC-1α protein and mRNA analysis, and IHC. **(A)** Retinal lysates collected from each group were probed for PGC-1α protein using capillary electrophoresis. No difference across groups for PGC-1α protein was observed (one-way ANOVA with Tukey’s multiple comparisons; *n* = 8 for Control and Control + OHT groups; *n* = 7 for KD and KD + OHT groups). **(B)** Quantitative PCR for the PGC-1α transcript (*Ppargc1a*) from whole retina mRNA indicated no difference in gene expression across all groups (one-way ANOVA with Tukey’s multiple comparisons; *n* = 9 for Control and Control + OHT groups; *n* = 6 for KD group; *n* = 4 for KD + OHT group). **(C)** To examine PGC-1α protein specifically in the ganglion cell layer, retinas were immunolabeled for PGC-1α, and the optical density of the immunolabel was quantified. There was no significant difference across all groups (Brown-Forsythe and Welch one-way ANOVA with Dunnett’s multiple comparisons; *n* = 2 for each group). **(D)** Representative cross-sections of the inner retina showing immunolabel for PGC-1α (red) and RGCs (RBPMS, green). White arrow in the KD retinal section points to an RBPMS-positive RGC that colocalized with PGC-1α. Scale bar = 20 μm.

Next, we endeavored to understand if the promotion of mitophagy would be balanced with a concomitant increase in mitochondrial biogenesis, as measured by transcriptional transactivator and mitochondrial biogenesis regulator PGC-1α. In whole retinal lysates, we measured PGC-1α protein (Figure 7A), finding no statistical differences across groups. Using mRNA isolated from the whole retina, we undertook quantitative RT-PCR for PGC-1α transcripts, also finding no change across the Control, Control + OHT, KD, or KD + OHT groups (Figure 7B). Recognizing that our mitolysosome analysis had focused on RGCs and Müller glia, not the whole retina, we immunolabeled the retina using an antibody against PGC-1α and colabeled with RBPMS for RGCs. An optical density analysis of PGC-1α in RBPMS-positive RGCs showed no significant differences across groups (Figure 7C). Figure 7D shows examples of immunolabeling for PGC-1α (red) and RBPMS (green) in each control and experimental group. Colocalization of PGC-1α with RBPMS was rare, with just one incidence shown in Figure 7D, in the section from the KD group (white arrow).

### Investigation of the BNIP3-NIX mitophagy pathway

**Figure 8:**
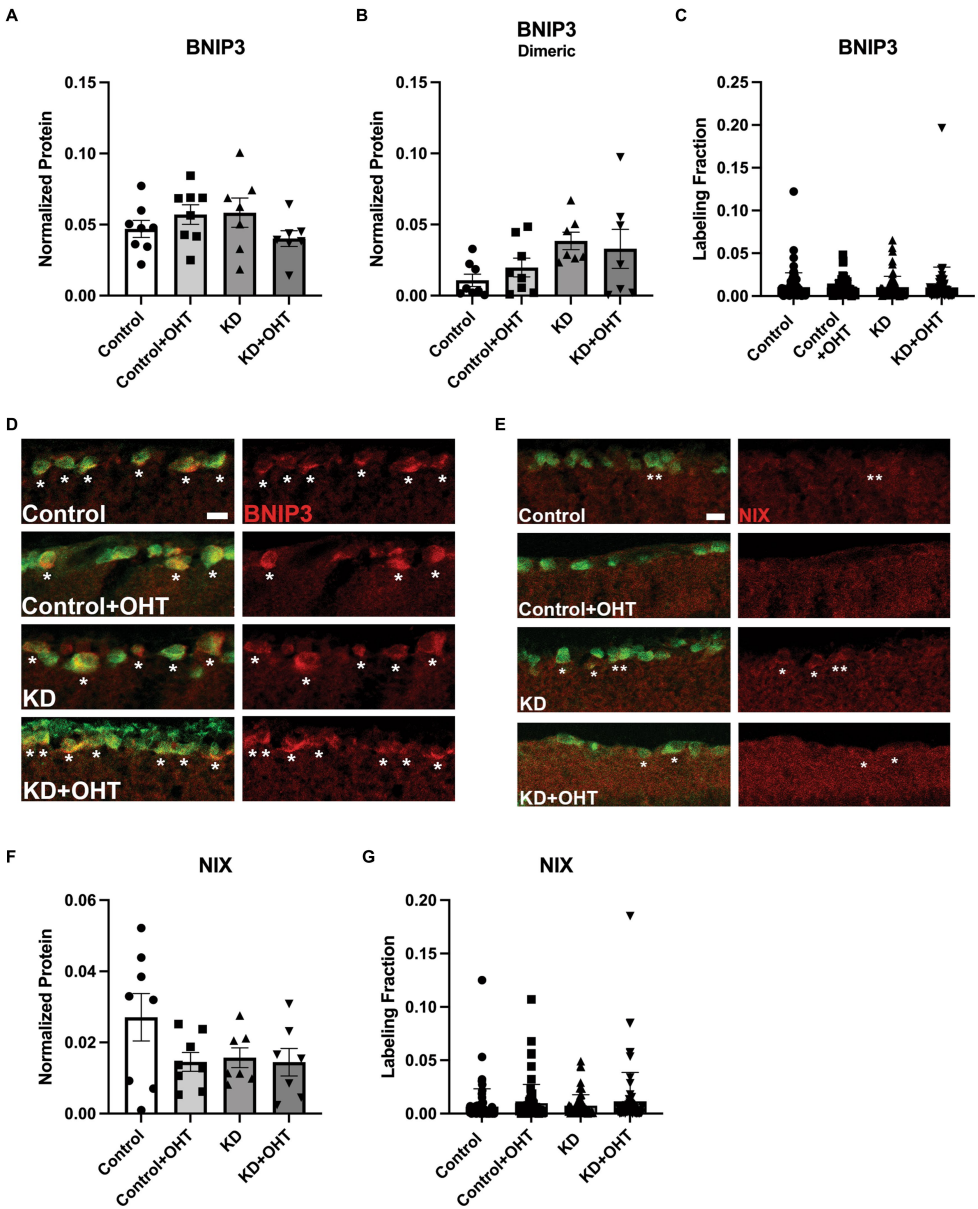
BNIP3 protein and mRNA analysis; NIX protein analysis. **(A)** Retinal lysates probed for BNIP3 in its monomeric or dimeric **(B)** form using capillary electrophoresis showed no statistical differences across groups (one-way ANOVA with Tukey’s multiple comparisons; *n* = 8 for Control and Control + OHT groups; *n* = 7 for KD and KD + OHT groups). **(C)** Retinal sections immunolabeled for RBPMS (green) and BNIP3 were analyzed for the labeling density in the ganglion cell layer. There were no statistical differences across groups (Brown-Forsythe and Welch one-way ANOVA with Dunnett’s multiple comparisons; *n* = 2 fo r each group). **(D)** Representative cross-sections of the inner retina showing immunolabel for BNIP3. Asterisks denote RGCs that have BNIP3 immunolabel. Many, but not all, RGCs were BNIP3-positive. Scale bar = 20 μm. **(E)** Representative cross-sections of the inner retina showing immunolabel for NIX. Asterisks denote RGCs that have NIX immunolabel. There were no NIX-positive RGCs in the Control + OHT group. Scale bar = 20 μm. **(F)** NIX protein in whole retinal lysate showed no statistical differences across groups (one-way ANOVA with Tukey’s multiple comparisons; *n* = 8 for Control and Control + OHT groups; *n* = 7 for KD and KD + OHT groups). **(G)** Retinal sections immunolabeled for RBPMS and BNIP3 were analyzed for the labeling density in the ganglion cell layer. There were no statistical differences across groups (Brown-Forsythe and Welch one-way ANOVA with Dunnett’s multiple comparisons; *n* = 2 for each group).

Having observed that hypoxia can be a stimulus for mitophagy in our model of OHT, we tested whether the BNIP3-NIX pathway was activated in RGCs where we had observed significant increases in mitolysosome density. BNIP3 is a member of the BH3-only subfamily whose expression is promoted by HIF-1 transcriptional activity; it localizes to the outer mitochondrial membrane after cellular stress (Mellor and Harris, 2007). BNIP3 and NIX act as mitophagy receptors that can tether the isolation membrane to mitochondria (Yamashita et al., 2024). Our analysis of whole retinal lysate yielded no changes in BNIP3, BNIP3 dimer, or NIX protein levels (Figures 8A,B,F). In order to refine our analysis, we immunolabeled the retina with BNIP3 or NIX and then measured the fraction of labeled tissue across our control and ketogenic diet groups, with or without OHT. We also observed no significant differences across groups when the immunolabeling analysis was restricted to the ganglion cell layer (Figures 8C,G). However, BNIP immunolabel (Figure 8D) was prominent in the RGCs, as denoted by asterisks. Many, but not all, RGCs contained BNIP3 immunolabel. For NIX immunolabeling (Figure 8E), colocalization with RGCs was observed in the Control, the KD, and the KD + OHT groups, but not in Control + OHT (asterisks).

## Discussion

Our goal with this study was to determine if the ketogenic diet promoted mitophagy since we had shown in a previous study that the diet was an effective promoter of mitochondrial biogenesis (Harun-Or-Rashid et al., 2018). We were able to demonstrate increased mitophagy with the ketogenic diet as well as with OHT in both RGCs and Müller glia. Another aim of the study was to determine if hypoxia was a stimulus for mitophagy in RGCs. Using pimonidazole, we were able to show greater mitolysosome number in Pimo-positive RGCs after OHT and in the ischemia–reperfusion-positive controls. Finally, we did not confirm that our observed mitophagy was a result of the BNIP3-NIX receptor-mediated pathway, suggesting alternate mechanisms of mitochondrial quality control predominated in this context.

### Ketogenic diet promotes mitophagy

Mitolysosome count was increased in the KD group compared to the Control in both RGCs and Müller glia, providing evidence that the ketogenic diet can stimulate mitophagy. In our previous study using the DBA/2 J mouse model of glaucoma, we showed that forcing cells to rely on ketones to produce cellular energy resulted in upregulation of PGC-1α and mitochondrial biogenesis (Harun-Or-Rashid et al., 2018). We theorized that the homeostatic balance of mitochondria would require increased mitophagy to accompany this upregulation in mitochondrial biogenesis. In skeletal muscle, SIRT1 is thought to promote mitochondrial biogenesis through the deacetylation of PGC-1α in fasting conditions and promote mitophagy by targeting mitophagic machinery (Palikaras et al., 2015). AMPK has also been shown to promote biogenesis through the regulation of PGC-1α (Palikaras et al., 2015), while mitophagy is upregulated by the enhancement of mitochondrial fission and TBK1-activated autophagosome engulfment in a PINK1-Parkin-independent pathway (Seabright et al., 2020). The ketogenic diet could play a role in SIRT1 activity through increased NAD^+^, which is a required substrate for SIRT1 (Maalouf et al., 2007; McCarty et al., 2015). There is also a possible pathway in which SIRT1 can promote AMPK activation through TBK1 (Lan et al., 2008). In addition to these effects, the ketogenic diet may be promoting mitophagy through upregulation of HIF-1α despite normoxic conditions, as exhibited in investigations of rat brains on this diet, therefore leading to transcription of BNIP3 and BNIP3L (NIX) (Puchowicz et al., 2008; Xu et al., 2010).

### Elevated intraocular pressure promotes mitophagy

Our results indicate that glaucomatous conditions can increase mitophagy, as demonstrated by significant increases in mitolysosomes in the Control + OHT mice compared to our control (naïve) group. This is an interesting find because there have been several studies proposing impaired mitophagy as a contributor to glaucomatous pathology and an avenue for therapeutic intervention (Dai et al., 2018; Hu et al., 2018; Hass and Barnstable, 2019). One study elucidated a link between mutations in optineurin with pathology of primary open-angle glaucoma, emphasizing evidence of impaired mitophagy leading to the accumulation of damaged mitochondria (Shim et al., 2016). In DBA/2 J mice, increased levels of mitochondria with altered morphology and diminished capacity for oxidative metabolism, together with decreased LAMP1, despite increased numbers of autophagosomes, led to the belief that these mice had impaired mitophagy (Coughlin et al., 2015). In the optic nerves of ocular hypertensive rats, mitophagosome numbers increased 3 days after elevated IOP, but within 2 weeks, these numbers decreased, leading to the belief that mitophagy may be upregulated to combat stress, but with chronic elevation, mitophagic mechanisms fail and may become impaired (Dai et al., 2018). Our analysis was of one moment in time, 4 weeks after OHT, where we observed significant increases in mitophagy over control. There can be a number of possible explanations for these findings of increased mitophagy when other investigations have data suggesting the opposite. For one, we used the MitoQC mouse, an animal enabling strong, direct *in vivo* evidence of mitophagy—mitochondria have green and red fluorescence that turns to red fluorescence alone when the mitochondrion is engulfed in a lysosome (McWilliams et al., 2016). We evaluated mitophagy 4 weeks after the commencement of OHT, which may represent an achievement of mitochondrial homeostasis that did not exist at other times post-OHT. Many of these other studies, including one of our own (Coughlin et al., 2015), provided only circumstantial evidence of mitophagy. Additionally, other studies showed that promotion of mitophagy was neuroprotective (Dai et al., 2018; Hu et al., 2018; Hass and Barnstable, 2019), which does not rule out the existence of an already increased mitophagy level; it would just demonstrate that more mitophagy under those degenerative conditions was also beneficial. A case in point is our original ketogenic diet study, in which we showed RGC structural and functional protection with the diet (Harun-Or-Rashid et al., 2018). In the current study, we demonstrated that the ketogenic diet had an additive effect on mitolysosome production, suggesting that increasing mitophagy beyond what was promoted with OHT contributed to better outcomes for the RGCs. Without further investigation into the quality of the unrecycled mitochondria, we cannot know for certain whether mitophagy is fully functional or impaired within the glaucomatous retinas of our study; however, in alignment with the above-mentioned studies, we do show increased mitolysosomes, indicative of increased mitophagy.

### Different metabolic needs between Müller glia and RGCs

Müller glia are the predominant glial cell type in the retina and are oriented radially to allow interaction across the entire retina (Vecino et al., 2016). These cells play a crucial role in retinal homeostasis by providing support for ion and water homeostasis, maintenance of the blood-retinal barrier, release of neurotrophic factors for survival, maintenance of retinal structural stability, provision of metabolic substrates, and more (Toft-Kehler et al., 2018). One suggestion of the metabolic substrate these cells can provide comes from the lactate shuttle hypothesis, which proposes that glial cells like astrocytes and Müller glia can uptake glucose and convert it to lactate to provide a metabolic substrate to RGCs (Hurley et al., 2015). Müller glia have a large concentration of mitochondria in their basal endfeet that are in close contact with RGCs (Choi et al., 2020). In light of the metabolic support these cells can provide to RGCs, we wanted to examine whether the ketogenic diet would impact mitophagy in these cells. The ketogenic diet and elevated IOP produced similarly increased mitophagy in Müller glia, indicating that the ketogenic diet was not as additive for promoting mitophagy as it was in the RGCs. Nevertheless, these results provided evidence to support that the diet does not have the same effect on mitochondrial homeostasis in RGCs and Müller glia. Müller glia show preference for glycolysis despite aerobic or anaerobic conditions (Winkler et al., 2000; Toft-Kehler et al., 2018). There is evidence that during inhibition of glycolysis, through loss of exogenous glucose, Müller glia rely on mitochondrial respiration and that simultaneous deprivation of oxygen and glucose is required to impact ATP production in these cells (Winkler et al., 2000). In addition to the flexibility these cells have for metabolic shift to maintain ATP production despite environmental conditions, there is evidence of Müller glia storing and metabolizing glycogen (PérezLeón et al., 2013). This provides a mechanism by which these cells maintain a backup supply of metabolic energy sources that can be utilized in low glucose conditions (Rombaut et al., 2023). Due to this, it is thought that Müller glia are less sensitive to acute metabolic deficits than RGCs (Silver et al., 1997). Considering the numerous mechanisms that Müller glia can utilize to maintain metabolic support, it appears that glaucomatous conditions are a sufficient stimulus to upregulate mitochondrial homeostatic processes, diminishing the potential for the ketogenic diet to induce metabolic shifts.

In contrast, within RGCs, the ketogenic diet promoted mitophagy to a greater degree than elevated IOP alone, as evidenced by a significant increase in mitolysosome number within the KD and KD + OHT groups compared to Control + OHT. Due to their sensitivity to metabolic deficits (Inman and Harun-Or-Rashid, 2017; Casson et al., 2021), RGCs may be more inclined to upregulate mitophagy via the ketogenic diet so that they may sustain mitochondrial homeostasis. The ketogenic diet does require RGCs to utilize ketone bodies for fuel, which obligates mitochondrial respiration and the attendant ROS generation that oxidative phosphorylation entails. The additional demand of the diet on the mitochondria may be the stimulus that promotes mitophagy beyond that initiated by OHT.

### Hypoxia as a driver for mitophagy

Through the ischemia–reperfusion experiments, we demonstrated that hypoxia provides a stimulus for mitophagy within the early stages of glaucomatous pathology (24 h after induction of elevated IOP). This was seen through increased mitolysosome number in mice with OHT (OHT) compared to our Naive (naïve) mice. In addition to this, our positive control mice showed statistically similar results as the OHT mice, suggesting that the additional IOP elevation (a difference of ~20 mmHg) did not further promote mitophagy. There could be a threshold of mitochondrial stress at which mitophagy is engaged, but at levels that can manage a wide range of elevated IOP and attendant hypoxia. As discussed above, there is evidence that increased mitophagy in early glaucomatous pathology could be a mechanism to adjust to stress produced by elevated IOP. Furthermore, hypoxia may be the stimulus leading to upregulation of mitophagy in the Control + OHT groups in the ketogenic diet experiments. Our pimonidazole experiment measured hypoxia-induced upregulation of mitophagy after 24 h in contrast to 4 weeks after OHT, yet we have evidence that hypoxic changes are likely a factor in longer-term glaucoma pathology. Pseudohypoxia, a phenomenon originally observed in diabetic conditions, refers to a metabolic state resembling hypoxia despite normal levels of oxygen (Williamson et al., 1993). A previous study revealed a link between pseudohypoxia and disrupted mitochondrial homeostasis in DBA/2 J mice (Jassim et al., 2021). In a more recent study, we confirmed chronic HIF-1α stabilization in DBA/2 J mice could induce a pseudohypoxic state and, by doing so, promote hypoxia-induced mitophagy through elevated protein levels of BNIP3 (Nsiah et al., 2023). Previously, our lab demonstrated hypoxia and increased *Hif-1a* transcripts in the retina 4 weeks after OHT, accompanied by the induction of autophagy (Jassim and Inman, 2019).

Interestingly, prominent pimonidazole staining seemed to be concentrated in the retinal ganglion cell layer and inner nuclear layer of the retinal sections. Our previous investigations have shown evidence of hypoxia in both RGCs and Müller glia after 4 weeks of OHT through pimonidazole localization (Jassim and Inman, 2019). Using a transgenic fluorescent reporter approach, we also observed HIF-1α stabilization after OHT in RGCs and Müller glia, with the highest reporter tdTomato labeling appearing at 6 h and 3 days (Jassim et al., 2022). The same study revealed Müller glia showed the most prominent tdTomato labeling (Jassim et al., 2022). Our relative lack of Müller glia labeling 24-h post-OHT likely reflects the lesser sensitivity of pimonidazole to HIF-1α stabilization/hypoxia as compared to the genetically encoded reporter construct (Butt et al., 2021; Jassim et al., 2022).

### Protein, mRNA, and Immunolocalization

For potential confirmation of mitochondrial biogenesis balanced with our evidence of increased mitophagy through the ketogenic diet, we analyzed the protein and mRNA of PGC-1α within the whole retina lysate, which showed no significant difference among the four groups. Since our mitolysosome quantification was focused within immunolabeled RGCs or Müller glia, we decided to analyze the localization of PGC-1α immunohistochemical staining within the inner retinal layer, but these results also showed no significant difference. These findings contrast with our observations in a previous study in the DBA/2 J mouse model of glaucoma, where we observed that the ketogenic diet afforded neuroprotection of RGCs concomitant with PGC-1α upregulation (Harun-Or-Rashid et al., 2018). The discrepancy could be a result of the duration of increased IOP (4 weeks in the current study, while 4+ months in the DBA/2 J), the length of time on the ketogenic diet (5 weeks vs. 8 weeks), or even mouse strain (C57Bl/6 J vs. DBA/2 J). Observing significant mitophagy at 4 weeks after OHT yet unchanged PGC-1α, as we do here, suggests there may be a means by which a cell could maintain mitochondrial number without biogenesis. Analyses of mitochondria in the DBA/2 J mouse have indicated that RGC axons have steady-state numbers of mitochondria, but that those organelles are significantly smaller with age and increased IOP (Coughlin et al., 2015). In fact, glaucoma significantly reduces the volume of mitochondria per axon in the RGC axon (Kleesattel et al., 2015). Fission is one way to increase mitochondrial number, and increased fission has been observed in the DBA/2 J (Ju et al., 2008). In cancer cells, upregulation of mitochondrial dynamics can maintain mitochondrial homeostasis (Towers et al., 2021). Mitochondria undergo fission prior to mitophagy in order to be small enough for engulfment, so there is a possibility that the mitochondria undergoing fission may ultimately be destined for mitophagy. Limits to the amount of mitophagy that can take place at any one time may create a pool of smaller mitochondria with indeterminate functional capability. Little is understood about the feedback mechanisms for mitochondrial dynamics that inform biogenesis. Additional studies using the MitoQC mouse at both shorter and longer time frames than the current study, as well as an investigation of mitochondrial dynamics, should provide insight into these possibilities.

To assess whether hypoxia promoted mitophagy in the model of glaucoma used here and 4 weeks after OHT, we evaluated the BNIP3-NIX pathway of receptor-mediated mitophagy. Protein changes in BNIP3 and NIX (BNIP3L) in whole retinal lysate or through immunolabeling of the inner retinal layer resulted in no global quantitative differences in BNIP3 or NIX, nor when analysis focused on the RGCs. This argues against receptor-mediated mitophagy with the ketogenic diet or after 4 weeks of elevated IOP from the microbead model. BNIP3 is a target of HIF-1α, and its phosphorylation mediates mitophagy by enabling direct binding of BNIP3 to LC3 (He et al., 2022). However, we did find significant colocalization of BNIP3 in the RGCs across all groups, and we also observed NIX colocalization in each group except the Control + OHT retina. We did not examine BNIP3 phosphorylation state nor c-Jun N-terminal kinase 1/2, the kinase that targets BNIP3, which could provide insight and potential confirmation of our results in a future investigation. In our previous study that stabilized HIF-1α in the retina for 4 weeks, we did observe a significant increase in both BNIP3 and NIX (Nsiah et al., 2023). One potential explanation for the discrepancy here is that our model of OHT results in sporadic HIF-1α and hypoxia (Jassim and Inman, 2019), while HIF-1α stabilized using Roxadustat was a much more powerful stimulus for BNIP3 and NIX expression (Nsiah et al., 2023). These data imply that context likely dictates the mechanism of mitophagy, and that it can vary. The ketogenic diet might have also played a role in specifying the exact mechanism of mitophagy shown here. Alternatively, the lack of an apparent role for BNIP3 and NIX may indicate a mitochondrial quality control mechanism in play that is not mitophagy at all. For example, mitochondrial-derived vesicles, which contain mitochondrial membrane and could thus be studded with GFP/mCherry fluorescence in our mice, are released by mitochondria and carry oxidized protein to lysosomes (Neuspiel et al., 2008; Soubannier et al., 2012). Once engulfed in a lysosome, these mitochondrial-derived vesicles (MDVs) would appear as red puncta and thus be indistinguishable from a recycled mitochondrion. Ruling out MDVs as contributors to the mitolysosomes we observe would require future electron microscopic analysis.

## Conclusion

Both OHT and the ketogenic diet promote mitophagy in the RGCs and Müller glia of the mouse retina. For RGCs, where oxidative phosphorylation plays a larger role in metabolic homeostasis than in Müller glia (Nsiah and Inman, 2022), the ketogenic diet promoted mitophagy beyond that which occurred with OHT. We also directly showed that cells exposed to acute hypoxia upregulate mitophagy. Though prominent in the retina from immunolabeling, BNIP was not quantitatively different across groups, suggesting the mechanism of mitophagy examined here may not have included the receptor-mediated form requiring BNIP-NIX activity.

## Data availability statement

The original contributions presented in the study are included in the article/supplementary material, further inquiries can be directed to the corresponding author.

## Ethics statement

The animal study was approved by Institutional Animal Care and Use Committee of the University of North Texas Health Science Center. The study was conducted in accordance with the local legislation and institutional requirements.

## Author contributions

AM: Data curation, Formal analysis, Investigation, Project administration, Writing – original draft. YF: Data curation, Investigation, Writing – original draft. DI: Conceptualization, Funding acquisition, Methodology, Resources, Supervision, Visualization, Writing – review & editing.
